# Quality improvement interventions in surgical oncology: systematic review of international studies

**DOI:** 10.1093/bjsopen/zrag053

**Published:** 2026-05-20

**Authors:** Adil Rashid, Sugeeta Sukumar, Joanna Dodkins, Georgia Zachou, Nicola S Fearnhead, Kate Walker, Ajay Aggarwal

**Affiliations:** National Cancer Audit Collaborating Centre, Clinical Effectiveness Unit, Royal College of Surgeons of England, London, UK; Department of Health Services Research and Policy, London School of Hygiene and Tropical Medicine, London, UK; National Cancer Audit Collaborating Centre, Clinical Effectiveness Unit, Royal College of Surgeons of England, London, UK; Department of Health Services Research and Policy, London School of Hygiene and Tropical Medicine, London, UK; National Cancer Audit Collaborating Centre, Clinical Effectiveness Unit, Royal College of Surgeons of England, London, UK; Department of Health Services Research and Policy, London School of Hygiene and Tropical Medicine, London, UK; National Cancer Audit Collaborating Centre, Clinical Effectiveness Unit, Royal College of Surgeons of England, London, UK; Department of Health Services Research and Policy, London School of Hygiene and Tropical Medicine, London, UK; Department of Colorectal Surgery, Cambridge University Hospital NHS Foundation Trust, Cambridge, UK; National Cancer Audit Collaborating Centre, Clinical Effectiveness Unit, Royal College of Surgeons of England, London, UK; Department of Health Services Research and Policy, London School of Hygiene and Tropical Medicine, London, UK; Department of Health Services Research and Policy, London School of Hygiene and Tropical Medicine, London, UK; Guy’s Cancer Centre, Guy’s and St Thomas’ NHS Foundation Trust, London, UK

**Keywords:** practice variation, quality assurance, surgical outcome, healthcare disparity

## Abstract

**Background:**

Substantial variation in surgical care and outcomes has been identified by quality assurance initiatives. Quality improvement (QI) interventions have the potential to address these disparities. This paper aimed to assess the types of QI interventions that have been designed, implemented, and evaluated in surgical oncology.

**Methods:**

A systematic search of MEDLINE and EMBASE was conducted to identify studies on QI interventions published between January 2000 and September 2025. Studies reporting the impact of the QI intervention on predefined quality deficits in clinical outcomes or care and process measures were selected. Data on study design, QI methodology, quality deficits, intervention types, and outcomes were extracted. Results were summarized using narrative synthesis and appraised using the Cochrane Effective Practice and Organization of Care risk of bias tool.

**Results:**

Of 11 373 studies, 109 were included. The majority were in the USA (48 (44.0%)), followed by Canada (20 (18.3%)), and the UK (11 (10.1%)). The commonest tumour types were gynaecological (22 (20.2%)) and colorectal (20 (18.3%)). The commonest quality deficits addressed were postoperative complications (22 (20.2%)) and prolonged length of stay (12 (11.0%)). One study was conducted globally, 6 nationally, 20 regionally, and 82 locally. Among randomized clinical trials, only 2 of 12 (16%) demonstrated a positive effect compared with 86 of 97 (89%) non-randomized studies. Only 28 (25.7%) studies referenced specific QI methodologies, most commonly the Plan–Do–Study–Act cycle (10 (9.2%)). QI interventions encompassed care pathway standardization, perioperative care bundles, audit, and feedback, combined with surgical skills workshops and digital tools, including checklists, and care coordination initiatives. Most studies were uncontrolled before-and-after studies (74 (67.9%)) and were classified as low quality.

**Conclusions:**

There are limited high-quality evaluations of QI interventions in the literature. Key gaps include interventions to improve equitable access to surgical care. Comprehensive QI studies leveraging large-scale, multidisciplinary collaborations and robust methodologies are needed to realize potential gains in surgical oncology care.

## Teaser

Substantial variation in surgical oncology care and outcomes have been identified and quality improvement interventions have the potential to address this. This systematic review highlights a lack of high-quality evaluations of QI interventions in the literature. Key gaps include interventions aimed at improving equitable access to surgical care.

## Introduction

There is growing emphasis on quality assessment (measuring quality) and quality assurance (QA) (identifying variation and its determinants) to stimulate improvements in quality of care^1,2^. QA can be used for institutional comparisons, outlier identification, and the exploration of practice variations^3^. With the exponential growth in electronic medical data, QA is increasingly delivered through national healthcare performance measurement activities and disease-specific registries^4^. In the USA, the American College of Surgeons maintains the National Surgical Quality Improvement Program^5^. In England and Wales, the National Cancer Audit Collaborating Centre (NATCAN) monitors care and outcomes across ten different cancer audits^6^.

QA projects have documented significant national and international variation in the quality of surgical care for patients with cancer. The National Prostate Cancer Audit^7–9^ showed substantial institutional variation in clinical outcomes in England and Wales, including rates of genitourinary complications and emergency readmissions following radical prostatectomy. Similarly, the Dutch Surgical Colorectal Audit^10^ identified wide variation in institutional anastomotic leak rates, even after adjustment for case-mix factors. The National Bowel Cancer Audit^11^ described considerable institutional variation in England and Wales in the proportion of patients with an unclosed diverting ileostomy 18 months after anterior resection for rectal cancer.

Quality improvement (QI) interventions have the potential to address these disparities. QI is a systematic, coordinated approach that supports healthcare professionals in identifying and solving problems affecting care quality, using specific methods and tools to achieve measurable improvement^12^.

Despite the growing interest in specific QI methods in the surgical setting^13,14^, there remains a limited understanding of the broader range of QI interventions used in surgical oncology and their efficacy. To address this gap, this review aims to evaluate systematically the types of QI interventions implemented in surgical oncology. Specifically, to assess the type, setting, methodology, and robustness of QI interventions, as well as their impact on care processes and clinical outcomes. Ultimately, this review aims to support healthcare professionals and stakeholders in delivering more effective QI interventions in surgical oncology, and therefore the focus is on studies that are designed robustly to support translation to other contexts.

## Methods

This systematic review was conducted according to the Cochrane Handbook for Systematic Reviews of Interventions^15^ and reported with reference to the PRISMA statement^16^. The protocol was registered in the PROSPERO database (registration number: CRD42024579971).

### Information sources and searches

Search terms were designed to identify QI interventions that specified a deficit in patient care within surgical oncology (*Appendix S1*). The authors conducted database searches of EMBASE and MEDLINE via the OVID platform. Studies published from January 2000 to September 2025 were included. After deduplication, records were uploaded to Rayyan^17^. The titles and abstracts were screened, and full-text articles were assessed against the inclusion criteria; discrepancies were resolved through consensus meetings with the senior author. Database searches were supplemented with hand-searching of references from included studies.

Studies were eligible if they prospectively evaluated a QI intervention in adult patients undergoing surgery for solid organ cancer.

Studies including surgery for both cancer and non-cancer indications were included if at least 50% of participants were undergoing cancer surgery. Interventions could be implemented at the local (within a single hospital/city), regional (within a single region), or national level. Randomized clinical trials (RCTs), cluster-randomized trials (cRCTs), controlled before-and-after studies (CBAs), interrupted time series (ITS), uncontrolled before-and-after studies (UCBAs), and cohort studies were included.

Publications were excluded if they were not written in English, involved patients younger than 18 years, or did not report baseline data, such as preintervention deficits or data from a parallel control cohort. Studies were also excluded if they focused solely on quality assessment or QA, assessed only adherence to QI interventions, or assessed only the implementation of best-practice guidelines. Studies were excluded if they were published as case reports, case series, meeting abstracts, letters, editorials, review articles, or in the ‘grey’ literature.

Data were extracted in Excel using predefined data extraction tables. Two authors performed data extraction. Discrepancies were resolved through discussions with the senior author. For each study, information on study design, country (classified as low- or middle-income country (LMIC) according to the Organization for Economic Co-operation and Development^18^), year of conduct, patient characteristics, study setting, and sample size was extracted. The impact of the QI interventions on the study’s primary outcomes was also extracted. The funding source was identified by explicit statements in the manuscript or the acknowledgement section. The funding classification was divided into government (for example, national government-level funding agency), industry (for example, pharmaceuticals), philanthropic (any charitable organization), individual cancer centre (if funded by a single cancer institute), and none stated^19^. The methodological framework that was used to develop the QI intervention, such as Plan–Do–Study–Act (PDSA) cycles^20^ or Lean methodology^21^, was also documented.

### Risk of bias assessment

Studies were appraised using the Cochrane EPOC risk of bias tool^22^ and Quality Improvement Minimum Quality Criteria Set (QI-MQCS)^23^. The QI-MQCS is a validated tool for appraising the quality of QI-specific aspects of QI studies. The appraisal tool includes 16 items, with each study scoring between 0 and 16. An online risk-of-bias tool was used for visual representation^24^. Risk of bias was not formally assessed for UCBA studies. The UCBA study design is at high risk of bias due to difficulties in establishing causality^25^.

### Outcomes of interest

The primary outcome was the impact of the QI intervention on the quality deficits (clinical outcomes or care process measures) for which the intervention was designed. Secondary analyses described study characteristics, including study design, country, tumour type, QI methodology, funding source, and the range of quality deficits targeted by the QI interventions.

### Statistical analysis

Counts and percentages were calculated for categorical variables. Due to methodological issues with UCBA studies, specifically the attribution of causation, a detailed evaluation of outcomes was not undertaken. A meta-analysis was not conducted due to heterogeneity in study design and outcomes.

## Results

**Fig. 1:**
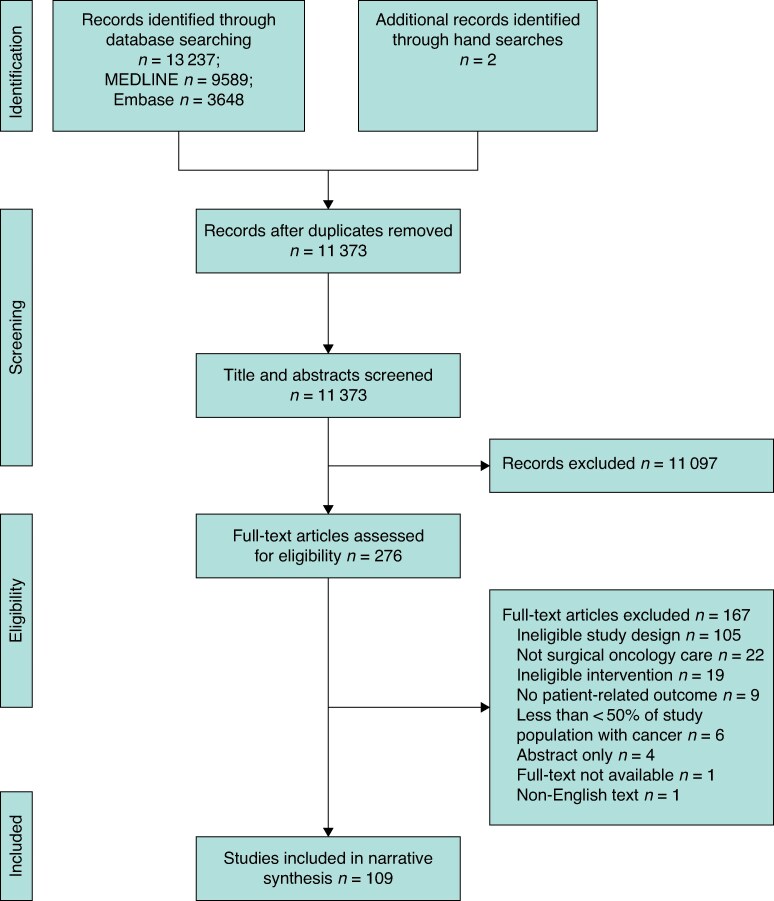
PRISMA 2009 flow diagram showing selection of articles for review. Database searches identified 13 237 citations, with two additional records found through hand-searching. After removal of duplicates, 11 373 titles and abstracts were screened, and 11 097 were excluded. The remaining 276 full-text articles were assessed, with 109 meeting the inclusion criteria

Database searches identified 13 237 citations, with two additional records found through hand-searching. After removal of duplicates, 11 373 titles and abstracts were screened, and 11 097 were excluded. Ninety-five per cent of these were excluded because they did not evaluate the implementation of a QI intervention in surgical oncology. These records were primarily observational studies focused on assessing associations with quality rather than evaluating interventions, clinical trials of radiotherapy or systemic anticancer therapies, and laboratory research. A smaller proportion was excluded due to publication type, including reviews, editorials, and clinical guidelines. The remaining 276 full-text articles were assessed, with 109 meeting the inclusion criteria (*Fig. 1*).

### Study characteristics

Across all 109 included studies (*Table 1* and *Table S1*), data from over 750 centres and over 140 000 patients were analysed. A total of 48 studies were conducted in the USA, 20 in Canada, 11 in the UK, 8 in the Netherlands, and 12 from the rest of Europe. Three studies were in LMICs countries, with two in Jordan^26,27^ and one in Pakistan^28^. In addition, 1 study was conducted globally, 6 studies were conducted nationally, 20 studies were conducted regionally, and 82 studies were conducted locally.

**Table 1:** Key characteristics of studies selected for the systematic review by study design

Study design	Unit	Cancer type	Patients	Centres	Quality deficit examples	QI intervention examples	QI methodology	Outcome of intervention	Funding
Cluster randomized clinical trials (*n* = 6)	Global (*n* = 1) National (*n* = 1) Regional (*n* = 4)	Colorectal (*n* = 3) Prostate (*n* = 1) Breast (*n* = 1) Lung (*n* = 1)	7586	494	Reduce anastomotic leak rates after right colectomy Improve perioperative nutritional management for older patients Increase proportion of patients who meet surgical lung cancer quality indicators	Behavioural change intervention covering surgical techniques, anastomotic leak risk assessment and safe anastomosis checklist Creation of an outreach team, geriatrician, and dietician Audit and feedback combined with educational workshops and intraoperative demonstrations	PRECEDE–PROCEED (*n* = 1) Capacity, Opportunity, Motivation, Behaviour model (*n* = 1) IHI collaborative model (*n* = 1) Not reported (*n* = 3)	Positive (*n* = 2) No effect (*n* = 4)	Received funding (*n* = 5) No funding (*n* = 1)
Randomized clinical trials (*n* = 6)	Regional (*n* = 3) Local (*n* = 3)	Multiple (*n* = 3) Breast (*n* = 2) Prostate (*n* = 1)	1174	13	Reduce SSIs Reduce postoperative delirium Improve urinary continence and sexual function after radical prostatectomy	SSI reduction bundle Preoperative comprehensive geriatric assessment Early physical activity intervention	Not reported (*n* = 6)	Positive (*n* = 0) No effect (*n* = 6)	Received funding (*n* = 4) No funding (*n* = 2)
Interrupted time series (*n* = 15)	National (*n* = 1) Regional (*n* = 2) Local (*n* = 12)	Gynaecological (*n* = 5) Breast (*n* = 3) Colorectal (*n* = 2) Multiple (*n* = 3) Brain (*n* = 1) Gastrointestinal (*n* = 1)	83 014	79	Reduce postoperative rates of VTE Improve preoperative biopsy rate Improve rate of same-day discharge for minimally invasive surgery	Postoperative VTE protocol Audit and feedback of surgeon performance Creation of standardized short-stay pathway that includes patient education	PDSA (*n* = 5)	Positive (*n* = 14) No effect (*n* = 1)	Received funding (*n* = 9) No funding (*n* = 6)
							DMAIC (*n* = 1) Root cause analysis (*n* = 1) Continuous QI (*n* = 1) Not reported (*n* = 7)		
Controlled before after study (*n* = 1)	National (*n* = 1)	Skin (*n* = 1)	Not reported	Not reported (2329 clinicians)	Reduce the overuse of Mohs micrographic surgery	Audit-and-feedback cycles with outlier doctors offered confidential mentoring	Not reported (*n* = 1)	Positive effect (*n* = 1) No effect (*n* = 0)	Received funding (*n* = 1) No funding (*n* = 0)
Cohort studies (*n* = 7)	Regional (*n* = 1) Local (*n* = 6)	Colorectal (*n* = 3) Brain (*n* = 1) Breast (*n* = 1) Lung (*n* = 1) Head and neck (*n* = 1)	3059	8	Improve multiple quality indicators for example length of stay, complications Improve patient selection for surgery Reduce local tumour recurrence	Prehabilitation intervention Creation of high-risk MDT meetings Audit and feedback for surgeons, focused on optimal surgical technique	PDSA (*n* = 1) Knowledge-to-action cycle (*n* = 1) Not reported (*n* = 5)	Positive (*n* = 3) No effect (*n* = 4)	Received funding (*n* = 5) No funding (*n* = 2)
Uncontrolled before after studies (*n* = 74)	National (*n* = 3) Regional (*n* = 10) Local (*n* = 61)	Gynaecological (*n* = 17) Colorectal (*n* = 12) Breast (*n* = 10) Urological (*n* = 10) Multiple (*n* = 9) Head and neck (*n* = 7) Lung (*n* = 4) Pancreas (*n* = 2) Oesophageal (*n* = 1) Liver (*n* = 1) Soft-tissue sarcoma (*n* = 1)	48 156	205	Reduce waiting times for surgical treatment Reduce geographical variation in resection rates for patients with colorectal liver metastases Improve multiple quality indicators (for example length of stay, mortality, complications).	Site-specific interventions included hiring surgeons, increasing operating theatre capacity, and improving appointment scheduling Standardized protocol for the surveillance, and referral of colorectal liver metastases to the central liver MDT Enhanced recovery after surgery pathway	PDSA (*n* = 4) Knowledge-to-action cycle (*n* = 2) Continuous QI (*n* = 2) DMAIC (*n* = 2) Lean (*n* = 1) 30-step-scenario (*n* = 1) CFIR (*n* = 1) Theoretical domains framework (*n* = 1) Value stream analysis (*n* = 1) Not reported (*n* = 59)	Positive (*n* = 68) No effect (*n* = 6)	Received funding (*n* = 33) No funding (*n* = 41)

QI, quality improvement; IHI, Institute for Healthcare Improvement; SSI, surgical site infection; PDSA, Plan–Do–Study–Act cycle; VTE, venous thromboembolism; DMAIC, Define, Measure, Analyse, Improve, and Control; MDT, multidisciplinary team; CFIR, consolidated framework for implementation research.

Of the included studies, 22 focused on gynaecological cancer, 20 on colorectal cancer, 17 on breast cancer, 15 on multiple cancer types, 8 on head and neck cancer, 6 on lung cancer, 5 on bladder cancer, and 5 on prostate cancer.

Twelve studies were RCTs, six of which were cRCTs. Half of the cRCTs utilized a stepped-wedge design, with the other half utilizing a parallel design. QI interventions included an online behavioural change intervention to reduce anastomotic leaks^29^, and creation of an outreach team, encompassing a geriatrician and dietician to improve guideline adherence^30^. Only 2 of the 12 studies (17%) demonstrated a positive effect on the primary outcome.

A total of 97 studies were non-randomized, including 15 ITS, 1 CBA, 7 cohort, and 74 UCBA studies. ITS QI interventions included audit-and-feedback cycles for surgeon performance^31^ and surgical-site infection reduction bundles^32,33^. Fourteen of the 15 ITS studies (93%) demonstrated a positive effect. One study used a CBA design to deliver a national audit-and-feedback intervention combined with mentoring, which reduced Mohs micrographic surgery overuse^34^. The most common QI intervention for UCBA studies was the implementation of Enhanced Recovery After Surgery (ERAS) care pathways (15 studies). In total, 68 of 74 (92%) UCBA studies demonstrated a positive impact.

Twelve out of 109 studies assessed audit and feedback of surgical quality, process of care, or clinical outcomes. Five of these studies combined audit and feedback with clinician education, which included practical workshops and cadaveric sessions on surgical techniques^35–39^. Feedback was provided through multiple modalities, including written report cards and verbal discussions with clinical leaders.

A total of 35 studies (32.1%) did not include a funding statement, and 17 (15.6%) reported that no funding was obtained for the study. In addition, 57 studies (52.3%) reported receiving funding, of which 3 had multiple funding sources. Most funding was from national governments (28 studies), with a further 13 studies funded by philanthropic sources, 7 by academic societies, and 6 by individual cancer centres.

### Study quality assessment and risk of bias

The quality of the 35 studies assessed using the QI-MQCS criteria^23^, excluding UCBA studies, was generally good, with scores ranging from 10 to 16 (*Table 2*). All studies reported on domains including organizational motivation, rationale for the QI intervention and its description, study design, comparator, and data source. Fewer studies reported organization characteristics that enabled readers to assess the generalizability to their organization. Eighteen studies (53%) reported spread, which addresses the intervention's replicability in other settings, and 12 (35%) studies reported penetration, which details the number of participating sites relative to the eligible number of sites.

**Table 2:** Quality improvement minimum quality criteria set quality assessment

	Quality improvement minimum quality criteria set domains																Total (row)
Author (year)	Organizational motivation	Initiative rationale	Initiative description	Organization characteristic	Implementation	Study design	Comparator	Data source	Timing	Adherence/fidelity	Health outcomes	Organizational readiness	Penetration/reach	Sustainability	Spread	Limitations	
Albertini *et al.* (2019)^34^	✔	✔	✔	✔	✔	✔	✔	✔	✔				✔	✔	✔	✔	13
Allard (2006) ^90^	✔	✔	✔	✔	✔	✔	✔	✔		✔	✔	✔	✔	✔	✔	✔	15
Badia *et al.* (2023)^33^	✔	✔	✔		✔	✔	✔	✔	✔	✔	✔	✔		✔	✔	✔	14
Boyle *et al.* (2023)^61^	✔	✔	✔	✔	✔	✔	✔	✔	✔		✔	✔		✔	✔	✔	14
Brown *et al.* (2018)^134^	✔	✔	✔		✔	✔	✔	✔	✔	✔		✔	✔	✔	✔	✔	14
Chahal *et al.* (2020)^50^	✔	✔	✔	✔	✔	✔	✔	✔	✔	✔	✔	✔		✔	✔	✔	15
Edwards *et al.* (2020)^135^	✔	✔	✔	✔	✔	✔	✔	✔	✔	✔		✔		✔		✔	13
ESCP EAGLE Safe Anastomosis Collaborative (2024)^29^	✔	✔	✔	✔		✔	✔	✔	✔	✔	✔	✔	✔	✔	✔	✔	15
Gilbert *et al.* (2021)^30^	✔	✔	✔	✔		✔	✔	✔	✔	✔	✔			✔	✔	✔	13
Guadagnoli *et al.* (2000)^136^	✔	✔	✔	✔	✔	✔	✔	✔	✔	✔				✔	✔	✔	13
Hempenius (2013)^44^	✔	✔	✔	✔	✔	✔	✔	✔	✔	✔	✔	✔	✔	✔		✔	15
Johnson *et al.* (2016)^32^	✔	✔	✔	✔	✔	✔	✔	✔	✔	✔	✔	✔		✔		✔	14
Khan *et al.* (2023)^79^	✔	✔	✔	✔	✔	✔	✔	✔	✔	✔	✔	✔				✔	13
Kim *et al.* (2022)^137^	✔	✔	✔	✔	✔	✔	✔	✔	✔	✔	✔	✔		✔		✔	14
Koinberg *et al.* (2006)^106^	✔	✔	✔		✔	✔	✔	✔	✔		✔	✔	✔		✔	✔	13
Kwaan *et al.* (2016)^45^	✔	✔	✔	✔	✔	✔	✔	✔	✔	✔	✔	✔		✔		✔	14
Latosinsky *et al.* (2007)^59^	✔	✔	✔		✔	✔	✔	✔	✔	✔			✔	✔	✔	✔	13
Lovrics *et al.* (2014)^31^	✔	✔	✔	✔	✔	✔	✔	✔	✔				✔	✔	✔	✔	13
McGinnis *et al.* (2022)^91^	✔	✔	✔	✔	✔	✔	✔	✔	✔	✔		✔	✔	✔		✔	14
McIsaac *et al.* (2022)^138^	✔	✔	✔	✔	✔	✔	✔	✔	✔	✔	✔	✔			✔	✔	14
Moore *et al.* (2017)^56^	✔	✔	✔	✔	✔	✔	✔	✔	✔	✔	✔	✔		✔	✔	✔	15
Nguyen *et al.* (2022)^51^	✔	✔	✔			✔	✔	✔	✔	✔	✔			✔	✔	✔	12
Nwaejike *et al.* (2016)^122^	✔	✔	✔			✔	✔	✔	✔		✔		✔	✔			10
Prescott *et al.* (2019)^40^	✔	✔	✔	✔	✔	✔	✔	✔	✔	✔		✔		✔		✔	13
Riblet *et al.* (2014)^60^	✔	✔	✔	✔	✔	✔	✔	✔	✔	✔		✔				✔	12
Russell *et al.* (2014)^58^	✔	✔	✔		✔	✔	✔	✔	✔	✔	✔	✔	✔		✔	✔	14
Simunovic *et al.* (2010)^104^	✔	✔	✔	✔	✔	✔	✔	✔	✔	✔	✔	✔	✔	✔	✔	✔	16
Simunovic *et al.* (2025)^38^	✔	✔	✔			✔	✔	✔	✔	✔	✔			✔		✔	11
Smithson *et al.* (2022)^89^	✔	✔	✔			✔	✔	✔	✔		✔			✔		✔	10
Spénard *et al.* (2025)^113^	✔	✔	✔	✔	✔	✔	✔	✔	✔	✔	✔	✔		✔		✔	14
van den Brink *et al.* (2007)^139^	✔	✔	✔	✔		✔	✔	✔	✔		✔			✔	✔	✔	12
Wang *et al.* (2019)^57^	✔	✔	✔		✔	✔	✔	✔	✔	✔	✔			✔		✔	12
Weber *et al.* (2011)^78^	✔	✔	✔	✔	✔	✔	✔	✔	✔		✔	✔		✔		✔	13
Wennerberg *et al.* (2023)^107^	✔	✔	✔	✔	✔	✔	✔	✔	✔	✔	✔					✔	12
Yuste Sanchez *et al.* (2015)^140^	✔	✔	✔		✔	✔	✔	✔	✔	✔	✔					✔	11
Total (column)	35	35	35	24	28	35	35	35	34	27	26	22	12	28	18	34	

ESCP EAGLE, European Society of Coloproctology safe-anastomosis programme in colorectal surgery.

RCTs were judged to be at low risk of bias across most domains, except for blinding, which was judged at high risk in 5 of 12 studies (*Fig. S1*). Due to the lack of randomization and the predominance of single-centre studies in CBA and cohort studies, the risk of bias was considered high or unclear (*Fig. S2*). In addition, four of these studies allocated the intervention to individuals within the same hospital, introducing a high risk of contamination. All ITS studies were judged as high or unclear risk in domain 1, which assesses whether the intervention is independent of other changes (*Fig. S3*). For example, one study^40^ acknowledged unaccounted confounders such as advances in surgical technique over the study period.

### Quality deficit addressed: the reduction of postoperative complications

Postoperative complications were the most frequently addressed quality deficit (22 studies), encompassing both procedure-specific complications (anastomotic leaks after colorectal surgery^29,41^, implant loss after immediate breast reconstruction^42^, and catheter-associated urinary tract infections after urological surgery^43^) and general complications (delirium^44^, surgical site infections (SSIs)^32,33,45–49^, venous thromboembolism (VTE)^39,50–55^, pulmonary complications^27,56^, and overall morbidity^57^). To reduce postoperative complications, interventions included a behavioural change strategy with online educational modules and a safe anastomosis checklist^29^, a multimodal delirium prevention bundle comprising a preoperative comprehensive geriatric assessment^44^, standardized SSI protocols including a change of instruments before wound closure^32,45,48,49^, risk-stratified VTE prophylaxis^50,52^, and perioperative pulmonary care bundles^27,56^.

### Quality deficit addressed: care pathway optimization

Twenty-two studies focused on improving care pathways by addressing multiple quality indicators throughout the patient journey^28,35,58–77^. Quality indicators reflected the diagnostic pathway (for example, preoperative imaging, biopsy rates, multidisciplinary team (MDT) discussions, and proportion seen by a clinical nurse specialist), and perioperative care (for example, timely surgery, quality of surgical resection, length of stay, postoperative complications, and functional outcomes). QI interventions frequently standardize care pathways. Examples of these pathways include multimodal prehabilitation^61,77^, standardized postoperative recovery protocols that set clear clinical goals and timelines^62,66,67,69,70^, and integrated coordination between primary and secondary care^74^. Three studies leveraged technology to standardize care pathways, using tools such as electronic medical record tracking^60^, electronic checklists^65^, and a web-based postoperative nursing pathway^63^. Audit and feedback strategies, combined with education or mentoring, were also used to benchmark hospital and surgeon performance across the patient pathway to identify and address opportunities for improvement^35,58,68,73^.

### Quality deficit addressed: reduction of postoperative length of stay

Twelve studies aimed to reduce postoperative length of stay. Eight of these used ERAS protocols, which included components such as early mobilization, pain control, nutrition, and VTE prevention^78–85^. Three studies standardized hospital discharge using clear clinical criteria, prescription protocols, and patient education^86–88^. One study implemented a mobile app for virtual monitoring of postoperative complications^89^.

### Quality deficit addressed: postoperative pain management

Twelve studies addressed deficits in postoperative prescribing and pain management^26,86,90–99^. Six of these used standardized multimodal analgesia protocols, often combined with patient and provider education to reduce excess opioid prescribing^86,92,94–97^.

### Quality deficit addressed: long-term outcomes

Twelve studies targeted longer-term postoperative outcomes, such as recurrence rates, functional recovery, and survival, through audit and feedback of surgical performance^38,100,101^, surgical skills workshops^102–104^, standardized treatment protocols^105^, and patient education focused on self-care and health promotion^106,107^.

### Quality deficit addressed: optimizing resource utilization

Eight studies addressed resource utilization^34,40,108–113^. Interventions included implementing evidence-based restrictive transfusion protocols, process mapping, and equipment optimization to shorten operative times.

### Quality deficit addressed: quality of surgical resection

Five studies addressed surgical resection quality (for example, surgical resection margin and lymph node yield) through audit-and-feedback cycles that reported surgeon performance, combined with clinician education, including surgical skills workshops^36,37,114,115^ and dual-surgeon operating^116^.

### Quality deficit addressed: waiting times and access to surgery

Four studies focused on reducing waiting times for surgery through the introduction of a nurse navigator^117–119^ and by combining audit and feedback with hospital-specific interventions, including expansion of the surgical workforce and operating theatre capacity, and streamlined appointment scheduling^120^. Three studies focused on increasing access to surgery through the introduction of a standardized protocol for referral to a central MDT^121^, creation of a high-risk MDT^122^, and the introduction of a structured, holistic assessment incorporating geriatric evaluation and targeted prehabilitation^123^.

### QI methodologies

Twenty-eight studies referenced specific QI or implementation methodologies. The most used methodology was the PDSA cycle (10). Three studies adopted the Define, Measure, Analyse, Improve, Control process, an advanced form of PDSA; three utilized continuous QI, which focuses on the progressive, incremental enhancement of processes, safety, and patient care; and three used the Knowledge-to-Action Framework. Other methodologies included Lean methodology, root cause analysis, the PRECEDE–PROCEED model, and value stream analysis.

### Outcomes of interventions

Eighty-eight (80.7%) studies reported statistical evidence of improvements following the QI intervention. The proportion of interventions that reported improvements varied by the quality deficit addressed, from 18 of 22 (82%) studies of postoperative complications, 18 of 22 (82%) studies of care pathway optimization, and 12 of 12 (100%) studies of length of stay.

## Discussion

This review synthesised 109 QI interventions in the field of surgical oncology. Interventions predominantly targeted quality deficits, including postoperative complications (20.2%), optimizing care pathways (20.2%), and postoperative length of stay (11.0%). Only three studies focused on waiting times for cancer surgery^117–119^ and two on equitable access to surgical care^121,122^; both have been identified as key priorities in global cancer care^124,125^. These systemic deficits, often linked to multifactorial determinants including resource constraints and social determinants of health^126^, may be underrepresented due to a bias favouring clinically modifiable process of care or clinical care measures over complex system-level defects requiring administrative or policy interventions. Similarly, only four QI interventions targeted traditional oncological outcomes such as local recurrence^102,104,105^ or long-term survival^101^, and four targeted the technical quality of surgical resection^36,37,114,115^. These deficits were typically addressed through practical educational workshops for surgeons, underscoring a gap in QI interventions supporting ongoing improvements in surgical technique.

Commonly utilized QI interventions included care bundles aimed at standardizing the delivery of care (for example, ERAS protocols and SSI prevention protocols), and audit-feedback loops combined with education or mentoring. Several studies leveraged ‘big data’ to monitor changes in performance and digital technology to deliver interventions through virtual follow-up and patient education.

Although the included studies collectively represent data from over 140 000 patients, the evidence base reveals several limitations. Notably, there is a predominance of single-centre studies, which, although offering valuable contextual insights into QI interventions delivered in specific institutional settings, limits the generalizability of findings. To evaluate the effectiveness and scalability of QI interventions across diverse healthcare environments more rigorously, greater emphasis on multicentre, nationally coordinated studies is required.

Another limitation of the current evidence base is the underrepresentation of studies conducted solely in LMICs (2.8%). Included studies are dominated by high-income countries, which may have limited applicability to healthcare systems with different resource constraints, infrastructure, and care delivery models. This imbalance may create substantial barriers to implementing effective QI interventions in LMICs. Given the lower cancer survival rates in LMICs^127^, which are linked to disparities in the quality of care, research that integrates QA and QI should be prioritized in LMICs.

Few studies developed a QI intervention utilizing established QI methodologies. Among studies that utilized a QI methodology, the most common was the PDSA model.

With 80.7% of QI interventions demonstrating an improvement in quality of care, QI interventions could play a key role in improving care. Nonetheless, the literature is dominated by single-centre non-randomized studies. When focusing on the highest quality of evidence, only 2 of 12 (17%) RCTs demonstrated a positive effect on the primary outcome. This may reflect difficulties in identifying the appropriate target for improvement or the key levers for change. Furthermore, this discrepancy may reflect publication bias, with a reluctance to publish lower-quality studies with ‘negative’ results. Notably, both positive RCTs targeted process-of-care deficits rather than direct clinical outcomes^30,58^, although such measures are recognized as key mediators of patient outcomes^128^.

This review also demonstrates that some well-intentioned improvement efforts at the local level are not replicated when subjected to more rigorous evaluation or scaled to multicentre settings^129^. For example, the positive outcomes observed with SSI-reduction bundles in single-centre UCBA studies^46,47^ were not replicated in a local RCT^45^ or in a national ITS study^33^. The success of local studies often reflects tailored adaptation to local contextual factors, including resource availability (for example, staffing ratios and equipment), institutional culture (for example, leadership engagement in QI), and workforce motivation. This contextual understanding may explain why evidence-based interventions fail when implemented elsewhere without similar adaptations. Furthermore, due to the high risk of bias^25^, UCBA studies have been recognized as overestimating the benefits of new interventions^130^.

Whereas methodologically rigorous designs, such as multicentre RCTs, are essential to establish causality, the success of QI interventions are context dependent^131^. Although the limited success of these studies may reflect an incomplete understanding of the quality deficit, it may also reflect the challenge of standardizing interventions that rely on local engagement and culture. Therefore, future QI efforts must balance methodological rigour with contextual understanding by developing adaptable intervention frameworks and employing pragmatic designs, such as cluster RCTs.

The safe-anastomosis programme in colorectal surgery (EAGLE) study^29^ demonstrated a ‘gold standard’ approach to QI by utilizing robust methodologies to design and implement a QI intervention^132,133^. Using a stepped-wedge cRCT design, the study assessed the causal impact of the intervention. The study, led by the European Society of Coloproctology EAGLE Safe Anastomosis Collaborative, involved multiple teams across low-, middle-, and high-income countries. A previous systematic review highlighted that, although QI collaboratives can be effective, their outcomes are often limited by weak study designs. Therefore, concerted efforts to combine QI collaboratives with robust methodology, as demonstrated by the EAGLE study, would strengthen the QI evidence base.

A limitation of this review is the inability to combine outcomes into a meta-analysis due to heterogeneity in study design and outcomes. Additionally, grey literature was excluded. Given that QI interventions are often mandated for healthcare professionals and that major surgical conferences feature dedicated QI sessions, there is likely a wide scope of QI work in the grey literature.

Overall, the current literature focuses on postoperative complications, care pathway optimization, and length of stay, with most studies conducted in Europe and North America targeting a narrow range of tumours. Key gaps include underrepresentation of LMICs and a lack of interventions addressing systemic challenges, such as waiting times and equitable access to surgical care. Although 80.7% of interventions reported improvements, most were poorly funded single-centre UCBA studies with limited generalizability. In contrast, more rigorous study designs, such as multicentre RCTs, demonstrated less favourable outcomes, suggesting that the success of local studies reflects adaptation to local contextual factors and highlighting challenges translating local successes to broader settings. To address quality deficits that extend beyond individual centres, robust study designs (for example, stepped-wedge cRCTs), supported by scalable tools such as national audits and multidisciplinary collaborations, combined with contextual understanding, are critical for designing and implementing interventions that reduce disparities in global cancer care.

## Supplementary Material

zrag053_Supplementary_Data

## Data Availability

The authors prepared a review protocol (PROSPERO Registration Number: CRD42024579971). The data and other material used for this systemic review are not publicly available but can be provided upon request.
