# Supplementary material for: Quality improvement interventions in surgical oncology: systematic review of international studies
Source: BJS Open. 2026 May 20;10(3):zrag053. doi: 10.1093/bjsopen/zrag053 (PMC13188151; doi:10.1093/bjsopen/zrag053)
Supplement: zrag053_Supplementary_Data [file zrag053_supplementary_data.docx]

**Quality improvement interventions in surgical oncology: a systematic review of international studies**

Adil Rashid^1,2^, Sugeeta Sukumar^1,2^, Joanna Dodkins^1,2^, Georgia Zachou^1,2^, Nicola S Fearnhead^3^, Kate Walker^1,2^, Ajay Aggarwal^1,2,4^

^1^Clinical Effectiveness Unit, Royal College of Surgeons of England, London, UK.

^2^Department of Health Services Research and Policy, London School of Hygiene and Tropical Medicine, London, UK.

^3^Department of Colorectal Surgery, Cambridge University Hospital NHS Foundation Trust, Cambridge, United Kingdom.

^4^Guy’s Cancer Centre, Guy’s and St Thomas’ NHS Foundation Trust, London, UK

**Corresponding author** Adil Rashid Email: [adil.rashid1@lshtm.ac.uk](mailto:adil.rashid1@lshtm.ac.uk) **ORCID ID**; 0000-0003-2371-093X **Twitter** @Mr_AdilRashid

**Supplementary Materials - Index**

| **Supplementary Appendixes** |  |
| --- | --- |
| Appendix 1 – Search terms Medline and Embase | *page 2* |
| **Supplementary Figures and Tables** |  |
| Supplementary Table 1 - Description of study design, quality improvement intervention methodologies and outcomes | *page 6* |
| Supplementary Figure 1 - Risk of bias for cluster randomised controlled trials and randomised controlled trials | *page 16* |
| Supplementary Figure 2 - Risk of bias for controlled before-after studies and cohort studies | *page 17* |
| Supplementary Figure 3 - Risk of bias for interrupted time series | *page 18* |

Appendix 1 – Search terms Medline and Embase

Search terms used in Medline. Search performed 03/02/2025 and rerun 23/12/2025.

| 1 | Quality of Health care/ or exp Quality assurance, health care/ |
| --- | --- |
| 2 | Quality Improvement/ |
| 3 | Total quality management/ |
| 4 | (quality adj (improvement or assurance or management or initiative*)).ti,ab,kw,kf. |
| 5 | (process adj management).ti,ab,kw,kf. |
| 6 | (improvement adj initiative*).ti,ab,kw,kf. |
| 7 | (best practice or benchmark*).ti,ab,kw,kf. |
| 8 | 1 or 2 or 3 or 4 or 5 or 6 or 7 |
| 9 | surgical oncology/ |
| 10 | (oncolog* adj3 (surg* or operative or operation or operations)).ti,ab,kw,kf. |
| 11 | 9 or 10 |
| 12 | (cancer* or neoplas* or metasta* or tumo?r* or oncolog* or anti-cancer or anti-neoplas* or anti-tumo?r*).ti,ab,kw,kf. |
| 13 | exp Neoplasms/ |
| 14 | 12 or 13 |
| 15 | exp Surgical procedures, Operative/ |
| 16 | (surg* or operative or operation or operations).ti,ab,kw,kf. |
| 17 | 15 or 16 |
| 18 | 14 and 17 |
| 19 | 11 or 18 |
| 20 | (outcome* or impact* or evaluat* or implementation).ti,ab,kw,kf. |
| 21 | diffusion of innovation/ or implementation science/ |
| 22 | Health plan implementation/ |
| 23 | exp Outcome assessment, health care/ |
| 24 | exp Program evaluation/ |
| 25 | 20 or 21 or 22 or 23 or 24 |
| 26 | 8 and 19 and 25 |
| 27 | child/ or child, preschool/ or infant/ or infant, newborn/ or infant, large for gestational age/ or infant, low birth weight/ or infant, small for gestational age/ or infant, very low birth weight/ or infant, extremely low birth weight/ or infant, postmature/ or infant, premature/ or infant, extremely premature/ or adolescent/ |
| 28 | pediatrics/ or neonatology/ or pediatric emergency medicine/ or perinatology/ |
| 29 | (p?ediatric* or child* or baby or babies or infant* or toddler* or neo nat* or neo-nat* or neonat* or newborn* or new-born* or preschool* or pre-school* or schoolchild* or school-child* or school-age* or underage* or under-age* or boy* or girl* or kid* or preadolescen* or pre-adolescen* or preteen* or pre-teen* or puber* or pubescen* or pre-pubescen* or juvenil* or adolescen*).ti,ab,kw,kf. |
| 30 | 27 or 28 or 29 |
| 31 | 26 not 30 |
| 32 | Animals/ not (Animals/ and Humans/) |
| 33 | 31 not 32 |
| 34 | editorial/ |
| 35 | news/ |
| 36 | exp historical article/ |
| 37 | anecdotes as topic/ |
| 38 | case reports/ |
| 39 | (letter or comment*).ti. |
| 40 | (abstract or comment or conference or letter).pt. |
| 41 | 34 or 35 or 36 or 37 or 38 or 39 or 40 |
| 42 | 33 not 41 |
| 43 | limit 42 to (english language and yr="2000 -Current") |

Search terms used in Embase. Search performed 03/02/2025 and rerun 23/12/2025.

| 1 | health care quality/ or benchmarking/ or exp health care surveillance/ or exp performance measurement system/ or "root cause analysis"/ |
| --- | --- |
| 2 | total quality management/ |
| 3 | (quality adj (improvement or assurance or management or initiative*)).ti,ab,kw,kf. |
| 4 | (process adj management).ti,ab,kw,kf. |
| 5 | (improvement adj initiative*).ti,ab,kw,kf. |
| 6 | (best practice or benchmark*).ti,ab,kw,kf. |
| 7 | 1 or 2 or 3 or 4 or 5 or 6 |
| 8 | surgical oncology/ |
| 9 | (oncolog* adj3 (surg* or operative or operation or operations)).ti,ab,kw,kf. |
| 10 | 8 or 9 |
| 11 | (cancer* or neoplas* or metasta* or tumo?r* or oncolog* or anti-cancer or anti-neoplas* or anti-tumo?r*).ti,ab,kw,kf. |
| 12 | exp neoplasm/ |
| 13 | 11 or 12 |
| 14 | exp surgical technique/ |
| 15 | (surg* or operative or operation or operations).ti,ab,kw,kf. |
| 16 | 14 or 15 |
| 17 | 13 and 16 |
| 18 | 10 or 17 |
| 19 | (outcome* or impact* or evaluat* or implementation).ti,kw,kf. |
| 20 | diffusion of innovation/ |
| 21 | implementation science/ |
| 22 | exp outcome assessment/ |
| 23 | exp program evaluation/ |
| 24 | 19 or 20 or 21 or 22 or 23 |
| 25 | 7 and 18 and 24 |
| 26 | exp child/ or juvenile/ |
| 27 | exp adolescent/ |
| 28 | exp pediatrics/ |
| 29 | (p?ediatric* or child* or baby or babies or infant* or toddler* or neo nat* or neo-nat* or neonat* or newborn* or new-born* or preschool* or pre-school* or schoolchild* or school-child* or school-age* or underage* or under-age* or boy* or girl* or kid* or preadolescen* or pre-adolescen* or preteen* or pre-teen* or puber* or pubescen* or pre-pubescen* or juvenil* or adolescen*).ti,ab,kw,kf. |
| 30 | 26 or 27 or 28 or 29 |
| 31 | 25 not 30 |
| 32 | letter/ or case report/ or case study/ |
| 33 | (letter or comment*).ti. |
| 34 | (abstract or comment or letter or editorial or note).pt. |
| 35 | 32 or 33 or 34 |
| 36 | 31 not 35 |
| 37 | limit 36 to (human and english language and yr="2000 -Current") |

**Supplementary Figures and Tables**

Supplementary Table 1 - Description of study design, quality improvement intervention methodologies and outcomes

| Author (Year, Country) | Unit | Cancer type | Patients | Centres | Quality deficit | Description of intervention | QI methodology | Outcome of intervention | Funding |
| --- | --- | --- | --- | --- | --- | --- | --- | --- | --- |
| Cluster Randomised Controlled Trials (cRCT) | | | | | | | | | |
| Brown et al (2018, Australia) | Regional | Prostate | 1071 | 9 | Increase post-operative referrals for consideration of adjuvant radiotherapy | Clinician education, to reinforce clinical practice guidelines, and promote practice change. Quarterly feedback of referral rates and a ‘flagging’ process by pathology services to identify high-risk patients. | PRECEDE-PROCEED planning model | No effect  32% of patients in the intervention-phase were referred compared with 30% in the control-phase (aRR = 1·06; 95% CI 0.74 to 1.51; P = 0·879) | Government and philanthropic |
| ESCP EAGLE Collaborative (2024, UK) | Global | Colon | 3039 | 355 | Reduce anastomotic leak rates after right colectomy for cancer and non-cancer pathology. | Behavioural change intervention delivered via online educational modules covering surgical techniques, risk-assessment for anastomotic leak and in-theatre safe anastomosis checklist. | MRC framework for complex interventions and the Capacity, Opportunity, Motivation, Behaviour model. | No effect  Anastomotic leaks before and after the intervention were 10.1% and 9.6% (aOR = 0.87; 95% CI 0.59 to 1.30; P = 0.498). | Government, professional body and industry |
| Gilbert et al (2021, France) | Regional | Colorectal | 147 | 5 | Improve adherence  to international nutritional guidelines for older patients | Creation of an outreach team, encompassing a geriatrician and dietician, which reviewed patients and provided training to healthcare professionals | Methodology not reported | Positive effect  Guideline concordant nutritional management was 39.2% in the intervention group compared to 1.4% in the control group (P = 0.0002). | Government |
| Guadagnoli et al (2000, USA) | Regional | Breast | 2314 | 28 | Reduce the proportion of women who report that surgeons did not discuss surgical options preoperatively | Surgeons received baseline data on performance. Opinion leaders discussed possible intervention strategies. | Methodology not reported | No effect  The improvement in the intervention and control hospitals did not differ. | Funding not declared |
| Russell et al (2014, UK) | National | Lung | Not reported | 81 | Increase the proportion of patients who meet lung cancer quality indicators. | Reciprocal site visits to observe MDT meetings and discuss feedback on performance of process and outcome measures. QI facilitators provided support on delivering a structured QI plan for 12-months. | Institute for Healthcare Improvement collaborative model | Positive effect  Active treatment increased in the intervention group by 5.2% compared with 1.2% in the control group (P = 0.055.) | Government |
| Simunovic et al (2010, Canada) | Regional | Rectal | 1015 | 16 | Reduce rates of permanent  colostomy and local cancer recurrence | Educational workshops and intraoperative demonstrations. Audit and feedback of performance. | Methodology not reported | No effect  No difference in rates of permanent colostomy or local recurrence between the intervention and control groups. | Government |
| Randomised Controlled Trials (RCT) | | | | | | | | | |
| Allard et al (2006, Canada) | Regional | Breast | 117 | 4 | Improve pain management. | Nursing team provided sessions for patients on coping with a stressful healthcare experience using self-regulation theory. | Methodology not reported | No effect  No significant difference between intervention and control group in any dimensions of pain. | Funding not declared |
| Hempenius et al (2013, The Netherlands) | Regional | Multiple | 297 | 3 | Reduce the incidence of postoperative delirium in older patients undergoing elective cancer surgery. | Preoperative comprehensive geriatric assessment, creation of an individual treatment plan targeted at risk factors for delirium, and daily inpatient visits by a geriatric nurse. | Methodology not reported      . | No effect  No significant difference in incidence of delirium between the intervention and control group | Government |
| Kwaan et al (2016, USA) | Local | Multiple | 233 | 1 | Reduce SSIs. | Abdominal closure protocol which included redraping, sterile glove change, and sterile equipment change, before fascial closure. | Methodology not reported | No effect  Surgical site infection rate was 11.6% in intervention group compared to 12.4% in control group (P=0.85). | Funding not declared |
| McIsaac et al (2022, Canada) | Local | Multiple | 204 | 1 | Improve postoperative functional recovery in older adults with frailty undergoing cancer surgery (6-min walk test distance). | Prehabilitation: Home-based total-body exercise training program, consisting of strength training, aerobic exercise, and flexibility training three times per week. Healthy eating before surgery guide. | Methodology not reported | No effect  No significant difference in measures of functional recovery (P=0.486), between the two groups. | Academic society |
| Wennerberg et al (2023, Sweden) | Regional | Prostate | 170 | 3 | Improve urinary continence, sexual function, and self-care, 1 year following radical prostatectomy. | Web-based self-care support for pelvic floor exercises and physical activity. Aimed at increasing motivation by improving knowledge, skills, and confidence to manage self-care. | Methodology not reported | No effect  No significant difference between the groups in urinary continence (P=0.09) or sexual function (P=0.97). | Government and philanthropic |
| Yuste et al (2015, Spain) | Local | Breast | 153 | 1 | Improve health-related quality of life in women 1-year after breast cancer surgery. | Early postoperative physiotherapy intervention including an education program on factors that may trigger upper limb lymphoedema, pain and reduce shoulder movement. | Methodology not reported | No effect  Addition of early physiotherapy to the education program did not demonstrate a statistically significant change in quality of life. | Government |
| Interrupted Time Series (ITS) | | | | | | | | | |
| Badia et al (2023, Spain) | National | Colorectal | 37,849 | 55 | Reduce SSIs. | SSI reduction bundle: perioperative antibiotic prophylaxis, mechanical bowel preparation, minimally invasive surgery, and plastic wound retractor | Methodology not reported | Positive effect  SSI rate improved from 18.4% to 10.2% (OR=0.50, 95% CI 0.47–0.52). | Government |
| Chahal et al (2020, Australia) | Local | Multiple | 24,953 | 1 | Reduce post-operative rates of VTE. | Introduction of a risk-stratified venous thromboembolism protocol. | Plan-do-study-act | Positive effect  Relative risk reduction of 79% (P<0.005) in postoperative VTE rates. | Single cancer centre |
| Edwards et al (2020, USA) | Local | Colorectal | 160 | 1 | Increase the proportion of eligible patients undergoing surveillance colonoscopy within 1 year of colorectal cancer resection. | Virtual gastroenterology-led surveillance clinic. Patients electronically enrolled into the clinic after MDT discussion about eligibility. | Plan-do-study-act | Positive effect  The proportion of people who underwent surveillance endoscopy within 1 year improved from 30.6% to 50.0% (P=0.031). | Funding not declared |
| Johnson et al (2016, USA) | Local | Gynaecological | 825 | 1 | Reduce SSIs. | SSI bundle: sterile closing tray and glove change for fascial closure. | Define, Measure, Analyse, Improve, and  Control approach | Positive effect  SSI rate improved from 6.0% to 1.1% with a relative risk reduction of 83% (P<0.01). | Funding not declared |
| Kim et al (2022, Canada) | Local | Gynaecological | 202 | 1 | Improve the rate of same day discharge for minimally invasive surgery. | Pre- and post-operative patient education. Standardised postoperative prescriptions and discharge criteria. | Root cause  analysis to identify barriers and facilitators | Positive effect  Same day discharge rate increased from 29% to 75% (P<0.001). | Academic society |
| Latosinsky et al (2007, Canada) | Regional | Breast | 7,022 | Not reported | Improve quality indicators for surgical breast cancer care. | Implementation of the Canadian Clinical Practice Guidelines for Breast Cancer. | Methodology not reported | No effect  No significant differences in rates of breast-conserving surgery (P=0.09), or adequacy of axillary node dissection (P=0.54). | Funding not declared |
| Lovrics et al (2014, Canada) | Regional | Breast | Not reported | 12 | Improve rates of preoperative core biopsy in patients with early-stage breast cancer. | Repeated cycles of audit and feedback of surgeon performance. Workshops organised to discuss audit results. | Continuous quality improvement | Positive effect  Preoperative  core biopsy rate increased from 73% to 92%. | Academic society |
| McGinnis et al (2022, Canada) | Local | Multiple | 429 | 1 | Improve the proportion of patients prescribed extended VTE prophylaxis (eVTEp) following surgery for gynaecological, and hepatobiliary cancers. | Postoperative VTE protocol including, pre‐printed prescriptions, an extended VTE checklist, and patient and staff education. | Plan-do-study-act | Positive effect  Proportion prescribed eVTEp increased from 3% to 70%. | None |
| Moore J et al (2017, UK) | Local | Multiple | 801 | 1 | Reduce the incidence of postoperative pulmonary complications. | Enhanced recovery after surgery with specific pulmonary measures e.g. deep breathing exercises with incentive spirometers. | Methodology not reported | Positive effect  Postoperative pulmonary complications reduced from 18.7% to 8.7%. | Government |
| Nguyen et al (2022, Canada) | Local | Gynaecological | 534 | 1 | Reduce the rate of postoperative symptomatic pulmonary embolus. | Dual extended thromboprophylaxis protocol based on international guidelines. | Methodology not reported | Positive effect  Pulmonary emboli rates decreased from 5.1% to 0% (p=0.001). | None |
| Prescott et al (2019, USA) | Local | Gynaecological | 1281 | 1 | Reduce the number of inappropriate perioperative blood transfusions. | Development of hospital guidelines. Delivery of education sessions. Monthly morbidity and mortality to provide feedback. | Methodology not reported | Positive effect  Perioperative transfusion rate decreased from 24% to 11%. (P<0.001). | Government |
| Riblet et al (2014, USA) | Local | Brain | 139 | 1 | Address the acute health care needs of patients with glioma during the perioperative period measured using 10 quality indicators. | Creation of an electronic medical record list which identifies new patients, standardises postoperative medication order sets and ensures that follow-up is organised | Plan-do-study-act | Positive effect Proportion of patients who met 10 quality indicators increased from 63% to 85% (P=0.003). | Funding not declared |
| Spénard (2025, Canada) | Local | Gynaecological | 1073 | 1 | Increase the treatment rate of preoperative anaemia and reduce peri-operative transfusion rate. | Standardising and automating anaemia screening and referral to the blood management clinic, while reducing the wait time for initial bloodwork. | Plan-do-study-act and lean methodology | Positive effect  Treatment rates increased from 8% to 39% (p < 0.01). Screening increased from 2% to 87% (p < 0.01). Transfusion rates decreased from 20% to 12%. | Government |
| Wang et al (2019, China) | Local | Gastrointestinal | 7,209 | 1 | Reduce postoperative complications. | Implementation of the WHO 19-item surgical safety checklist. | Methodology not reported | Positive effect  Postoperative complications decreased from 16.3% to 14.3% (aOR 0.853, 95% CI=0.743–0.979, P=0.024). | Government |
| Weber et al (2011, USA) | Local | Breast | 537 | 1 | Reduce length of stay for patients undergoing unilateral mastectomy. | Enhanced short-stay pathway which includes standardised analgesia and antiemetic order sets, and patient education. | Methodology not reported | Positive effect  Proportion with 1-day length of stay increased from 9.6% to 82.7%. | Philanthropic |
| Controlled Before After Study (CBA) | | | | | | | | | |
| Albertini et  al (2019, USA) | National | Skin | Not reported | Not reported (2329 clinicians) | Reduce the overuse of Mohs micrographic surgery. | Confidential, benchmarked, personalised audit-and-feedback reports. Outlier doctors identified if mean number of procedures per patient > 2 standard deviations above the national mean. Outliers offered confidential mentoring. | Methodology not reported | Positive effect  Intervention outlier surgeons exhibited a mean reduction of 0.26 procedures per patient, compared to a 0.11 reduction in the control group outliers (P= 0.002). | Philanthropic |
| Cohort Studies | | | | | | | | | |
| Boyle et al (2023, UK) | Local | Colorectal | 94 | 1 | Improve postoperative care measured using multiple quality indicators e.g. length of stay, complication rates, and functional outcomes. | Trimodal prehabilitation intervention including:  1) Physical exercise programme  2) Nutritional screening and dietary plan by dietician  3) Psychological support | Plan–Do–Study–Act | No effect  No significant difference in clinical outcomes. Intervention group showed improved preoperative functional outcomes. | Single cancer centre |
| Khan et al (2023, USA) | Local | Brain | 520 | 1 | Reduce hospital length of stay after craniotomy. | Enhanced recovery after surgery pathway including early physical and occupational therapy sessions, and early surgical drain removal. | Methodology not reported | Positive effect Median length of stay was 1.93 days in intervention group compared to 2.92 in the control group (P < 0.001). | Funding not declared |
| Koinberg et al (2006, Sweden) | Local | Breast | 96 | 2 | Improve patient-reported long-term well-being. | Multidisciplinary educational programme for patients focused on self-care and health promotion, delivered by a specialist nurse, a physiotherapist, and social worker. | Methodology not reported | No effect  No difference in well-being. | Government |
| Nwaejike et al (2016, UK) | Local | Lung | 820 | 1 | Improve patient selection for surgery and clinical outcomes for high-risk patients. | High-risk multidisciplinary team meetings including surgeons, anaesthetists and specialist thoracic surgical nurses. | Methodology not reported | Positive effect  No significant differences in short-term mortality despite a higher predicted mortality rate in the HRMDT group. | Funding not declared |
| Simunovic (2025, Canada) | Regional | Rectal | 1080 | Not stated | Reduce local tumour recurrence. | Educational sessions, and audit and feedback for surgeons, focused on optimal surgical technique. | Knowledge-to-action cycle | No effect  No association with improved patient outcomes, including local tumour recurrence. | Government |
| Smithson et al (2022, USA) | Local | Colorectal | 265 | 1 | Reduce length of stay after ileostomy formation. | Mobile-based app that provides patients with preoperative, and post-operative education. The app includes daily questions regarding recovery, and advice for patients to contact their physician if any metrics suggest complications. | Methodology not reported | Positive effect Length of stay was significantly lower in the intervention group compared to the control (P < 0.001) with no increase in readmissions. | Government |
| van den Brink et al (2007, The Netherlands) | Local | Head and neck | 184 | 2 | Improve patient reported quality of life 3-months after surgery. | Electronic health information support system for patients during their first six weeks after discharge. | Methodology not reported | No effect  Compared to the control group, only one of 22 quality of life parameters significantly improved at 12 weeks. | Government |
| Uncontrolled Before After Studies (UCBA) | | | | | | | | | |
| Abdel Jalil et al (2021, Jordan) | Local | Multiple | 1665 | 1 | Reduce postoperative pulmonary complications. | Perioperative pulmonary care bundle, including smoking cessation, perioperative pulmonary interventions and early mobilization. | Methodology not reported | Positive | None |
| Aletti et al (2009, USA) | Local | Gynaecological | 237 | 1 | Reduce variation in quality of cytoreductive surgery (amount of residual disease after surgery). | Confidential audit and feedback to compare surgical performance. Educational seminars and cadaveric sessions on surgical techniques. | Methodology not reported | Positive | Funding not declared |
| Baack Kukreja et al (2017, USA) | Local | Bladder | 200 | 1 | Reduce postoperative length of stay. | Cystectomy enhanced recovery pathway including nutrition, hydration, VTE prevention, and pain control. | Methodology not reported | Positive | Funding not declared |
| Bakkum-Gamez et al (2011, USA) | Local | Gynaecological | 981 | 1 | Reduce variation in quality of pathological staging measured using number of lymph nodes present in surgical specimen. | Implementation of guidelines on surgical approach and periodic assessment and feedback of surgical quality. | Methodology not reported | Positive | Single cancer centre |
| Baliski et al (2014, Canada) | Local | Breast | 197 | 1 | Reduce waiting times for surgery. | Introduction of a nurse navigator facilitating tests and expediting surgical referrals. | Methodology not reported | Positive | Philanthropic |
| Bao et al (2016, China) | Local | Multiple | 1365 | 1 | Improve overall quality of cancer care measured using multiple quality indicators. | Clinical pathway that sets goals for patients. | Methodology not reported | Positive | Government |
| Bernard et al (2021, Canada) | Local | Gynaecological | 628 | 1 | Reduce postoperative length of stay. | Enhanced recovery after surgery pathway. | Plan-Do-Study-Act | Positive | Funding not declared |
| Bisch et al (2018, Canada) | Local | Gynaecological | 519 | 2 | Reduce postoperative length of stay. | Enhanced recovery after surgery pathway. | Methodology not reported | Positive | Funding not declared |
| Boitano et al (2020, USA) | Local | Gynaecological | 2549 | 1 | Reduce excess postoperative opioid prescribing. | Post-operative restrictive opioid prescribing algorithm and patient education. | Methodology not reported | Positive | Funding not declared |
| Bondeven et al (2020, Denmark) | Regional | Rectal | 627 | 2 | Reduce 3-year local cancer recurrence rate. | Multidisciplinary workshops with a focus on surgical techniques for clinicians. | Methodology not reported | Positive | Academic society |
| Bonkowski et al (2018, USA) | Local | Multiple | 88 | 1 | Improve postoperative pain management. | Pain management education for staff and development of operational guidelines. | Knowledge-to-action cycle | No effect | Funding not declared |
| Chang et al (2002, Taiwan) | Local | Kidney | 124 | 1 | Improve overall quality of postoperative cancer care measured using multiple quality indicators. | Web based care pathway utilised by nurses to record the daily patient progress. Pathway includes activity, nutrition, education, psychosocial support, and a discharge plan | Methodology not reported | Positive | Government |
| Chiang et al (2020, USA) | Local | Bladder | 319 | 1 | Reduce post-operative rates of VTE. | VTE prophylaxis program, including risk assessment tool, to identify patients at risk of post discharge VTE for extended prophylaxis. | Methodology not reported | Positive | None |
| de Groot et al (2014, The Netherlands) | Local | Gynaecological | 115 | 1 | Reduce post-operative length of stay. | Enhanced recovery after surgery pathway. | Methodology not reported | Positive | Funding not declared |
| de Kok et al (2010, The Netherlands) | National | Breast | 421 | 4 | Reduce post-operative length of stay, | Short stay admission pathway which included checklist-guided consultation between patient and breast nurse. | Methodology not reported | Positive | Funding not declared |
| De Pastena et al (2025, Italy) | Local | Pancreas | 1321 | 1 | Reduce SSIs. | Antimicrobial stewardship program, including  pre-operative screening, and tailored surgical  antibiotic prophylaxis. | Methodology not reported | Positive | Government |
| Du et al (2019, USA) | Local | Head and neck | 228 | 1 | Reduce excess postoperative opioid use. | Multimodal analgesia protocol. | Continuous quality improvement | Positive | None |
| Fearon et al (2020, USA) | Local | Multiple | 1031 | 1 | Reduce excess postoperative opioid prescribing. | Standardised opioid prescriptions. | Methodology not reported | Positive | Government |
| Frost et al (2025, UK) | Regional | Gynaecological | 178 | 2 | Increase the number of  patients receiving surgery and reduce inequalities due to age, frailty, and psychosocial problems. | Geriatric assessment and multimodal prehabilitation programme of physiotherapy, psychological, nutritional and functional support. | Plan-do-study-act | No effect | Philanthropic |
| Golisch et al (2025, USA) | Regional | Multiple | 2012 | 16 | Improve adherence to  guideline-recommended post-discharge VTE prophylaxis. | Peer coaching, educational materials for patients and clinicians including a toolkit, and comparative audit-and-feedback reports. | Methodology not reported | Positive | Funding not declared |
| Golshan et al (2016, USA) | Local | Breast | 99 | 2 | Reduce waiting times for mastectomy with immediate reconstruction. | Surgery Coordinator worked with breast and reconstructive surgical teams to identify the earliest surgery dates and facilitated operative bookings. | Define, measure,  analyse, improve, and control | Positive | Government |
| Greenberg et al (2020, USA) | Local | Bladder | 104 | 1 | Reduce excess postoperative opioid use. | Multimodal opioid-sparing pain regimen, and improved patient and staff education regarding non-opioid medications. | Methodology not reported | Positive | None |
| Hagan et al (2013, UK) | Local | Lung | 202 | 1 | Improve quality of surgery measured by anatomical resection and number of lymph nodes resected. | Audit and feedback of surgical quality, | Methodology not reported | Positive | Funding not declared |
| Haro et al (2021, USA) | Local | Lung | 295 | 1 | Reduce postoperative length of stay | Enhanced recovery after surgery pathway. | Methodology not reported | Positive | None |
| Hirschhorn et al (2014, Australia) | Regional | Prostate | 139 | 3 | Improve pre-operative uptake of pelvic floor muscle training (PFMT). | 1) Patient information guides 2) Evidence summary of evidence for urologists  3) Audit and feedback on provision/receipt of preoperative PFMT to clinicians  4) Provision of a directory of local providers of PFMT | Theoretical Domains Framework | Positive | Government |
| Hite et al (2021, USA) | Local | Breast | 119 | 1 | Reduce excess postoperative opioid prescribing. | Standardized prescribing, patient education, and an opioid disposal system | Methodology not reported | Positive | Funding not declared |
| Iqra et al (2023, Pakistan) | Local | Colorectal | 150 | 1 | Improve multiple quality indicators: post-operative length of stay and short-term complications. | Surgical site infection bundle and enhanced recovery after surgery | Methodology not reported | Positive | Funding not declared |
| Jansen-Landheer et al (2009, The Netherlands) | Regional | Soft tissue sarcoma | 159 | 12 | Improve guideline-concordant quality of care measured using quality indicators (e.g. MDT discussion, diagnostic imaging). | Establishment of national guidelines for management of patients with soft tissue sarcoma | Methodology not reported | Positive | Philanthropic |
| Kain et al (2021, USA) | Local | Head and neck | 76 | 1 | Improve overall quality of postoperative care measured using multiple quality indicators (e.g. wound complications, 30-day return to theatre). | Electronic medical record-based pre- and post-operative checklist that records the care of head and neck cancer patients undergoing microvascular free-tissue reconstruction. | Methodology not reported | Positive | None |
| Kalogera et al (2017, USA) | Local | Gynaecological | 386 | 1 | Reduce the rate of anastomotic leak. | Standardised protocol for when to perform temporary bowel diversion after rectosigmoid resection for cytoreduction. | Methodology not reported | Positive | Funding not declared |
| Kim et al (2021, Canada) | Local | Gynaecological | 193 | 1 | Reduce excess postoperative opioid prescribing. | Restrictive opioid prescription protocol with multimodal analgesia, and patient and provider education. | Methodology not reported | Positive | Government |
| Kiong et al (2021, USA) | Local | Head and neck | 400 | 1 | Improve multiple quality indicators (e.g. complications, length of stay, and readmission rates). | Enhanced recovery after surgery pathway. | Methodology not reported | Positive | Philanthropic |
| Knight et al (2020, UK) | Local | Breast | 160 | 1 | Reduce implant loss following immediate implant-based breast reconstruction. | Intervention bundle, including more than 25 protocol changes e.g. antibiotic protocol, implant handling and outpatient follow up. | Methodology not reported | Positive | Funding not declared |
| Koh et al (2011, USA) | Local | Breast | 55 | 1 | Improve timely access to care for patients with breast cancer. | Oncology nurse navigator who acts as the care coordinator, ensuring that patients have timely access to their first consultation with breast cancer specialists. | Methodology not reported | Positive | Funding not declared |
| Kumar et al (2021, Canada) | Local | Prostate | 422 | 1 | Improve resection margin and urinary, and sexual outcomes 1-year following radical prostatectomy. | Audit and feedback to surgeons of oncological and functional outcomes via surgical report cards. | Methodology not reported | No effect | Philanthropic |
| Letton et al (2013, UK) | Local | Gynaecological | 110 | 1 | Improve multiple quality indicators (e.g. time taken to mobilise, length of stay). | Enhanced recovery integrated care pathway including time-specified parameters to be achieved in pre and postoperative days. | Methodology not reported | Positive | Funding not declared |
| Lewis et al (2015, USA) | Local | Head and neck | 4007 | 1 | Improve multiple quality indicators (e.g. length of stay, return to theatre,  30-day mortality, SSIs). | Individual surgeon risk-adjusted audit and feedback discussions. | Methodology not reported | Positive | None |
| Lippitt et al (2017, USA) | Local | Gynaecological | 219 | 1 | Reduce SSIs. | SSI prevention bundle including preoperative use of oral antibiotics and mechanical bowel preparation, and perioperative wound management. | Methodology not reported | Positive | Funding not declared |
| Mateshaytis et al (2024, Canada) | Local | Gynaecological | 100 | 1 | Improve rate of same day discharge. | Same day discharge established as the default discharge plan for patients undergoing minimally invasive hysterectomy, patient education. | Plan-do-study-act | Positive | Funding not declared |
| McKechnie et al (2022, Canada) | Local | Multiple | 525 | 1 | Reduce post-operative rates of VTE. | Extended VTE prophylaxis after abdominopelvic cancer surgery. | Methodology not reported | No effect | Academic society |
| McMullan et al (2025, UK) | National | Gynaecological | 55  Pre-intervention not reported | 3 | Improve multiple quality indicators (e.g. length of stay, complications). | Prehabilitation: smoking cessation, nutritional intervention, occupational therapy, and physical activity. | Methodology not reported | Positive | Philanthropic |
| Merolle et al (2020, Italy) | Local | Multiple | 600 | 1 | Reduce inappropriate perioperative blood transfusions. | Postoperative patient blood management bundle which included Points of Care tests for continuous monitoring of haemoglobin. | Methodology not reported | Positive | Government |
| Meyer et al (2018, USA) | Local | Gynaecological | 607 | 1 | Reduce excess postoperative opioid use. | Enhance recovery after surgery program. | Methodology not reported | Positive | Government |
| Moore C et al (2021, USA) | Local | Head and neck | 50 | 1 | Improve post-operative nutritional status. | Enhanced recovery after surgery nutrition protocol including preoperative nutritional assessment, nutritional education and nutritional supplements | Value stream analysis | Positive | Single cancer centre |
| Morse et al (2019, USA) | Local | Head and neck | 108 | 1 | Reduce intensive care use for patients undergoing head and neck microvascular reconstruction. | Postoperative clinical care pathway detailing timelines for patient care goals. | Methodology not reported | Positive | None |
| Nguyen et al (2019, Canada) | Local | Gynaecological | 563 | 1 | Reduce SSIs. | SSI prevention bundle: preoperative chlorhexidine shower, prophylactic antibiotics, glycaemic control, normothermia, and separate closing tray. | Methodology not reported | Positive | Funding not declared |
| Nolan et al (2019, USA) | Local | Breast | 102 | 1 | Increase the proportion of patients, in the surgical breast cancer clinic who smoke, referred to smoking cessation services. | Smoking cessation referral intervention including advice on benefits of quitting on cancer care, and referral to smoking cessation services on an opt-out basis. | Consolidated Framework for Implementation Research | Positive | Single cancer centre |
| Planas et al (2007, Spain) | Local | Colorectal | 549 | 1 | Improve post-operative nutritional status. | Local practice guidelines/specialised nutritional support team to recommend most beneficial type of nutritional support. | Methodology not reported | Positive | Government |
| Schmeler et al (2013, USA) | Local | Gynaecological | 664 | 1 | Reduce post-operative rates of VTE. | Standardized extended duration VTE prophylaxis guidelines | Methodology not reported | Positive | Single cancer centre |
| Shah et al (2021, USA) | Local | Head and neck | 169 | 1 | Reduce postoperative emergency department visits. | Telephone follow-up and virtual wound check within 72 hours of discharge. | Methodology not reported | Positive | None |
| Sims et al (2024, USA) | Local | Oesophageal | 296 | 1 | Improve multiple quality indicators (e.g. length of stay, return to theatre). | Enhanced recovery after surgery pathway. | Lean methodology and Gemba Walk method | Positive | None |
| Simunovic et al (2004, Canada) | Regional | Rectal | 120 | 3 | Reduce rate of permanent colostomy after rectal cancer surgery. | Clinician education workshops, and operative demonstrations. | Continuous quality improvement | Positive | Philanthropic |
| Smeltzer et al (2019, USA) | Regional | Lung | 2206 | 11 | Improve overall long-term survival. | Provided confidential benchmarked performance feedback of quality indicators to clinicians in interactive sessions. | Methodology not reported | Positive | Government |
| Sorenby et al (2019, Sweden) | Local | Bladder | 275 | 1 | Reduce rates of local recurrence after transurethral resection of bladder cancer (TURBT). | Standardised treatment protocol which included eight experienced surgeons to be responsible for all procedures and implemented use of photodynamic diagnosis-guided TURBT. | Methodology not reported | Positive | Government |
| Soria-Aledo et al (2011, Spain) | Local | Colorectal | 270 | 1 | Improve multiple quality indicators (e.g. length of stay, complications, mortality). | Clinical pathway which encompasses a time matrix containing medical and nursing processes for patient care (e.g. drain removal). | Methodology not reported | No effect | Philanthropic |
| Spencer et al (2019, USA) | Local | Urological | 107 | 1 | Reduce rates of catheter associated urinary tract infections in patients discharged with an indwelling urinary catheter. | Pre-operative patient education program for management of a catheter at home. Scheduling follow-up appointment for catheter removal prior to discharge. | Methodology not reported | Positive | None |
| Tanna et al (2023, USA) | Local | Breast | 181 | 1 | Reduce duration of breast cancer operations. | Created a process map outlining the overall flow of the operation and created specialised instrument trays. | Define, measure, analyse, improve and control | Positive | None |
| Teeuwen et al (2011, The Netherlands) | Local | Rectal | 123 | 1 | Improve multiple quality indicators (e.g. length of stay, mortality, complications). | Enhanced recovery after surgery pathway. | Methodology not reported | Positive | Funding not declared |
| Telfah et al (2015, Jordan) | Local | Multiple | 167 | 1 | Improve adherence to antimicrobial prophylaxis guidelines. | Updated institutional guidelines for surgical antimicrobial prophylaxis. Assignment of a clinical pharmacist to the surgical department. Staff education on antibiotic utilisation. | Methodology not reported | Positive | Funding not declared |
| Thompson et al (2025, USA) | National | Breast | Not reported | 62 | Reduce waiting times from biopsy to first treatment. | Comparative audit-and-feedback reports. Site-specific QI interventions included hiring  surgeons, increasing OR capacity, improving appointment scheduling, and workflow process. | Methodology not reported | Positive | None |
| Tiernan et al (2010, UK) | Regional | Colorectal | 362 | 5 | Reduce geographical variation in resection rates for patients with colorectal liver metastases. | Standardised protocol for the surveillance, and referral of colorectal liver metastases to the central liver MDT. | Methodology not reported | Positive | Funding not declared |
| Tremblay St-Germain et al (2017, Canada) | Local | Pancreas | 157 | 1 | Improve multiple quality indicators (e.g. length of stay, mortality, complications). | Enhanced recovery after surgery pathway. | Knowledge-to-action cycle | Positive | Academic society |
| Trocchia Mattessich (2020, USA) | Local | Colorectal | 104 | 1 | Reduce postoperative length of stay. | Bedside goals-to-discharge patient checklist for patients to note their postoperative real-time progress. | Methodology not reported | Positive | Funding not declared |
| Turrentine et al (2018, USA) | Local | Multiple | 6257 | 1 | Reduce post-operative rates of VTE. | VTE stratification tool embedded into electronic medical records, linking risk factors to VTE prophylaxis order sets. | Methodology not reported | Positive | None |
| van der Geest et al (2012, The Netherlands) | Regional | Colorectal | 1669 | 9 | Reduce variation between hospitals in quality indicators (e.g. proportion of patients treated within 5 weeks of first hospital visit). | Results of quality indicators fed back to hospitals annually. Results presented verbally and discussed within the multidisciplinary oncology team by a senior team member and improvement opportunities identified. | Methodology not reported | Positive | Philanthropic |
| Van Houdt et al (2013, Belgium) | Local | Prostate | 176 | 1 | Improve multiple patient reported quality indicators (e.g. physiotherapy consultation, pain management). | Care pathway to facilitate communication between primary and secondary care, standardised prescription forms and patient information pack. | 30-step-scenario | No effect | Philanthropic |
| Veerbeek et al (2011, The Netherlands) | Regional | Breast | 6858 | 9 | Reduce variation in quality indicators (e.g. waiting times for surgical treatment). | Hospitals received written feedback with benchmarked performance on indicators annually. Training sessions to discuss results with experts in the field which stimulated improvement initiatives. | Plan-Do-Study-Act cycle | Positive | Philanthropic |
| Wehry et al (2015, USA) | Local | Liver | 186 | 1 | Reduce inappropriate perioperative blood transfusions. | Restrictive blood transfusion guidelines. | Methodology not reported | Positive | Government |
| White et al (2023, USA) | Local | Colorectal | 715 | 2 | Reduce SSIs. | Colorectal Surgical Site Infection Prevention Bundle and Checklist (e.g. sterile instrument tray for fascial closure). | Methodology not reported | Positive | Funding not declared |
| Wichmann et al (2025, Australia) | Local | Colorectal | 387 | 1 | Improve multiple surgical outcomes focusing quality of surgical resection (e.g. lymph node yield, minimally invasive surgery, complications). | Dual surgeon operating. | Methodology not reported | Positive | Funding not declared |
| Wilks et al (2009, USA) | Local | Colorectal | 346 | 1 | Improve multiple quality indicators (e.g. surgical resection margin, use of neoadjuvant therapy). | Standardised suspected colorectal cancer clinical pathway including MDT meetings, dedicated physician assistants, clinic support staff, and weekly Morbidity and Mortality meetings. | Methodology not reported | Positive | Funding not declared |
| Wilson et al (2022, USA) | Local | Bladder | 101 | 1 | Reduce proportion of transurethral resection of bladder tumours that do not contain muscle. | Audit and feedback of surgical performance and clinician education. | Methodology not reported | Positive | None |
| Wu et al (2020, USA) | Local | Lung | 233 | 1 | Improve overall quality of care using guideline-based quality indicators. | Guidelines–Based Decision Support Tool. The interactive web-based tool allows patients to enter their clinical, radiographic, and pathologic characteristics and explore guideline-based treatment combinations. | Methodology not reported | Positive | Government |
| Zanolli et al (2023, USA) | Local | Gynaecological | 668 | 1 | Reduce postoperative opioid prescribing and improve pain management. | Postoperative opioid use predictive calculator. The model generates a recommended opioid prescription which informs the clinicians prescribing practice. | Methodology not reported | Positive | Academic society |

cRCT Cluster randomised controlled trial; RCT Randomised controlled trial; CBA Controlled before-after study; ITS Interrupted time series; UCBA Uncontrolled before-after study; SSI Surgical site infection; VTE Venous thromboembolism; QI Quality improvement; MDT Multidisciplinary team; RR Risk ratio; OR Odds ratio.

Supplementary Figure 1 - Risk of bias for cluster randomised controlled trials and randomised controlled trials


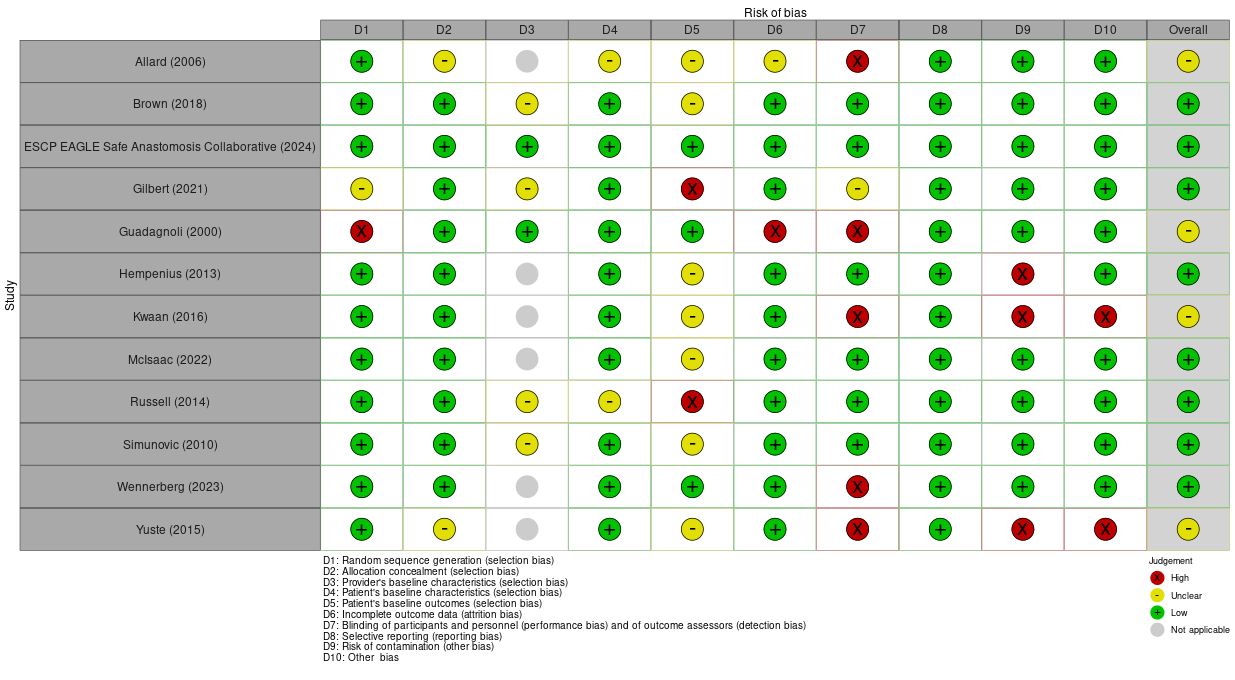


Supplementary Figure 2 - Risk of bias for controlled before-after studies and cohort studies

**
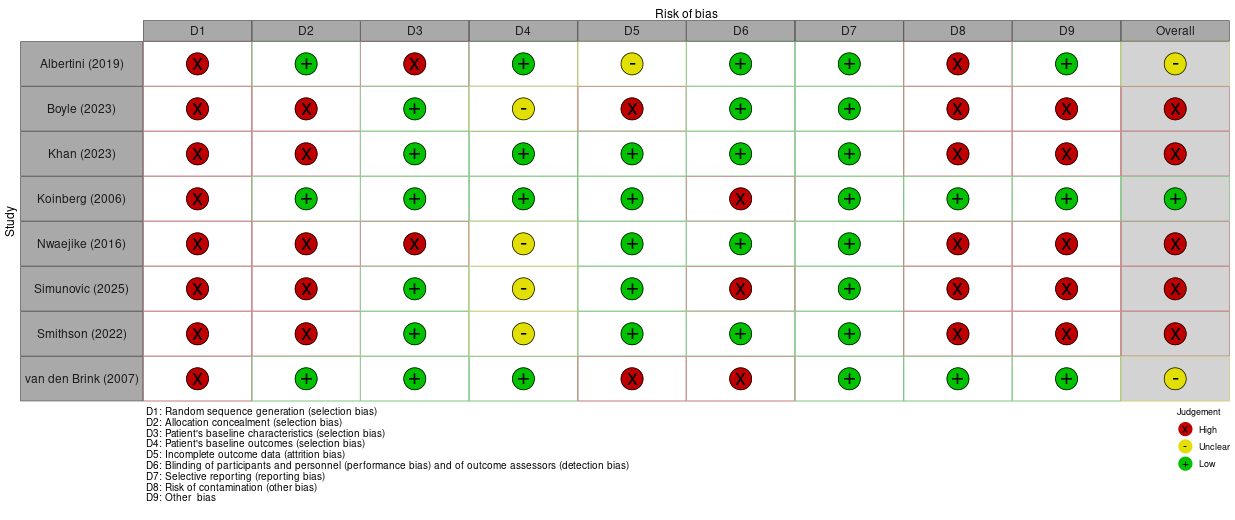
**

Supplementary Figure 3 - Risk of bias for interrupted time series

**
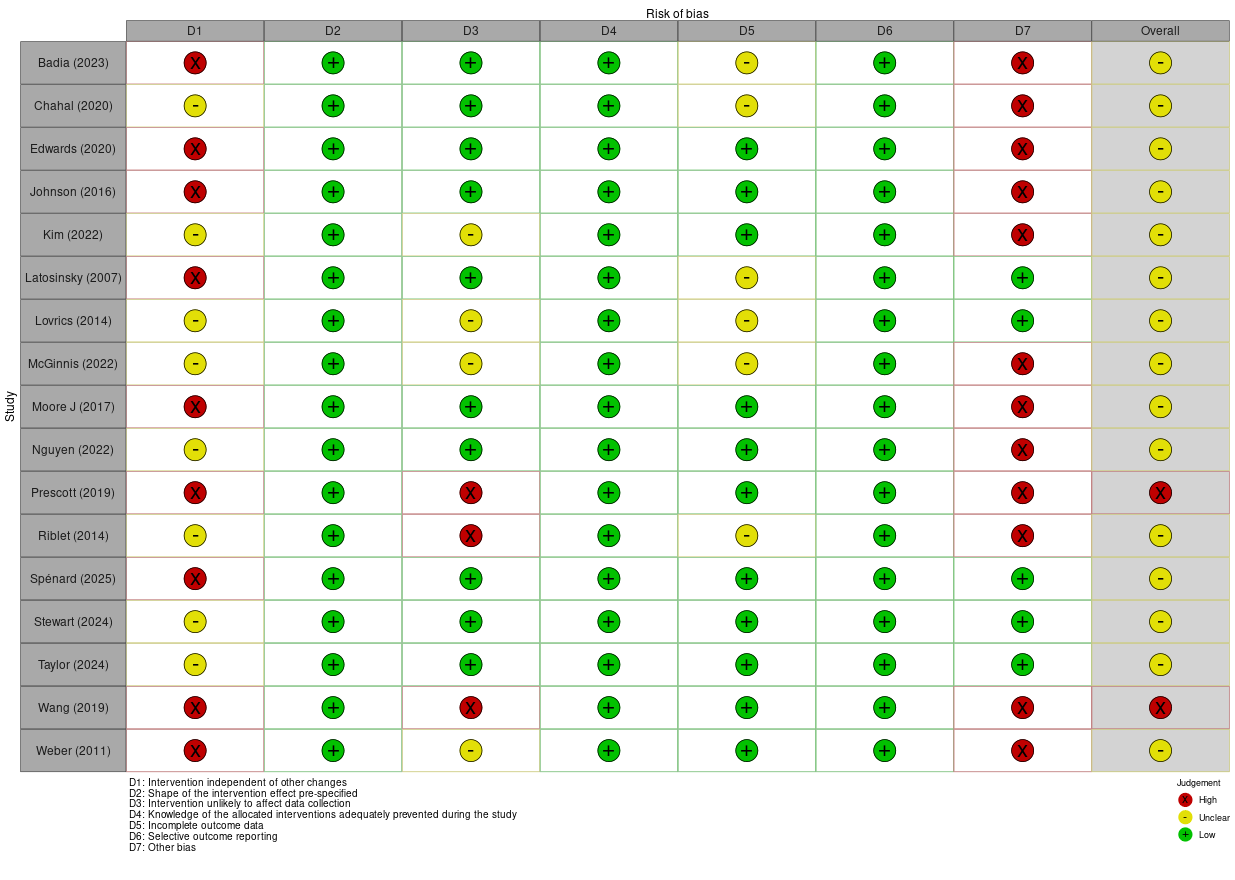
**
